# Revisiting Fruit Allergy: Prevalence across the Globe, Diagnosis, and Current Management

**DOI:** 10.3390/foods12224083

**Published:** 2023-11-10

**Authors:** Thanachit Krikeerati, Pongsawat Rodsaward, Jutamard Nawiboonwong, Kanokkarn Pinyopornpanish, Songwut Phusawang, Mongkhon Sompornrattanaphan

**Affiliations:** 1 Division of Allergy and Clinical Immunology, Department of Medicine, Faculty of Medicine Siriraj Hospital, Mahidol University, Bangkok 10700, Thailand; t.krikeerati@gmail.com; 2 Faculty of Medicine Siriraj Hospital, Center of Research Excellence in Allergy and Immunology, Mahidol University, Bangkok 10700, Thailand; 3 Division of Immunology, Department of Microbiology, Faculty of Medicine, Chulalongkorn University, Bangkok 10330, Thailand; pongsawat.r@chula.ac.th; 4 Division of Allergy and Clinical Immunology, Department of Medicine, Faculty of Medicine, Chulalongkorn University, Bangkok 10330, Thailand; 5 Department of Medicine, Faculty of Medicine, Khon Kaen University, Khon Kaen 40002, Thailand; j.nawib@gmail.com; 6 Division of Allergy and Clinical Immunology, Department of Medicine, Faculty of Medicine, Chiangmai University, Chiangmai 50200, Thailand; knk_04@hotmail.com; 7 Division of Allergy, Immunology and Rheumatology, Department of Medicine, Ramathibodi Hospital, Mahidol University, Bangkok 10400, Thailand; sphusawang@gmail.com

**Keywords:** anaphylaxis, fruit allergy, food allergy, food safety, food-dependent exercise-induced anaphylaxis, fruit hypersensitivity, gluten, IgE, oral food challenge, plant food allergies

## Abstract

Fruit allergies manifest with a diverse array of clinical presentations, ranging from localized contact allergies and oral allergy syndrome to the potential for severe systemic reactions including anaphylaxis. The scope of population-level prevalence studies remains limited, largely derived from single-center or hospital-based investigations. In this comprehensive review, we conducted a systematic literature search spanning the years 2009 to 2023, with full acknowledgment of potential analytical biases, to provide a global overview of fruit allergy prevalence. The primary mechanistic underpinning of fruit allergies stems from cross-reactivity between aeroallergens and food allergens, a consequence of structurally similar epitopes—a phenomenon recognized as pollen food allergy syndrome (PFAS). In the era of molecular allergology, numerous studies have dissected allergen components with substantial clinical relevance. Within this review, we explore important allergenic molecules found in plant-based foods, scrutinize pertinent cross-reactivity patterns, and offer insights into management recommendations. Additionally, we compare guideline recommendations to enhance clinical understanding and inform decision making.

## 1. Introduction

In recent decades, both children and adults globally have experienced a rise in food allergy prevalence rates. Factors such as heightened exposure to food allergens, early diagnostic recognition, and evolving environmental elements disrupting immune tolerance have played significant roles. Interestingly, the prevalence exhibits variation based on regional dietary practices. Notably, industrialized or westernized communities are more impacted than their agricultural or non-westernized counterparts, with the younger population being more susceptible than adults. Food allergy manifestations span a spectrum from mild to severe reactions and potentially life-threatening conditions such as anaphylaxis. While most food allergies stem from the production of and subsequent response to allergen-specific immunoglobulin E (IgE) against allergenic proteins, categorized as type I hypersensitivity reactions, a minority are non-IgE mediated.

In the field of food allergies, plant-based allergens emerge as the predominant culprits, often appearing during childhood but potentially manifesting in adulthood as well [1]. While plant-based foods, especially fruits and vegetables, are promoted for their health benefits and their role in preventing cardiovascular and metabolic diseases, some individuals with predispositions may face allergic reactions when consuming these foods. Allergens are present in the peel, pulp, and seeds of fruits. They can be found in an array of products including juices, pastries, sweets, preserves, and even as components in various recipes. Even without heat processing or pasteurization, merely cutting a fruit like an apple could cause the oxidative breakdown of its allergenic substances [1,2].

Allergic responses to fruits can present in two predominant clinical patterns: one that originates directly from the fruit and is not associated with pollen sensitivities, and another that is intricately linked to pollen allergies. The latter phenotype is attributed to the cross-reactivity between inhalant allergens and those found in foods. This phenomenon is underpinned by the generation of cross-reactive specific IgE (sIgE) antibodies, which identify and bind to structurally analogous allergenic epitopes, irrespective of the degree of taxonomic relation between their respective sources [3]. An illustrative example of such a cross-reactivity syndrome is pollen food allergy syndrome (PFAS). Within this category of secondary food allergies, the spectrum of allergic symptoms can range from localized reactions confined to the oropharynx (oral allergy syndrome, OAS) to severe systemic reactions such as anaphylaxis [4]. Accurate diagnosis requires a comprehensive medical history assessment, complemented by objective confirmation of sensitization through either skin tests or specific IgE testing. Understanding molecular sensitization patterns and host-specific factors holds promise for predicting the clinical severity of plant-based food allergies [5].

In this review, we aim to explore the prevalence of fruit allergies, examine the diverse clinical manifestations, and provide insights into clinical cross-reactivity, with a specific focus on commonly associated fruits. Additionally, we will discuss general management strategies for addressing plant-based food allergies in current recommendations.

## 2. Global Prevalence of Fruit Allergy

Our approach aimed to consider studies conducted within the last 15 years (from 2009 onwards) to ensure the most recent information, with an emphasis on nationwide surveys to represent national prevalence. However, in instances where national studies were lacking, we considered including single-center or hospital-based studies as well. We systematically searched PubMed using the term “fruit allergy[tw] AND prevalence OR prevalence [MeSH Terms]” to determine fruit allergy prevalence. Additionally, we employed the term “Food allergy[tw] OR ‘Food Hypersensitivity’ [MeSH Terms] AND prevalence OR prevalence [MeSH Terms]” to avoid potentially overlooked studies. 

**Figure 1:**
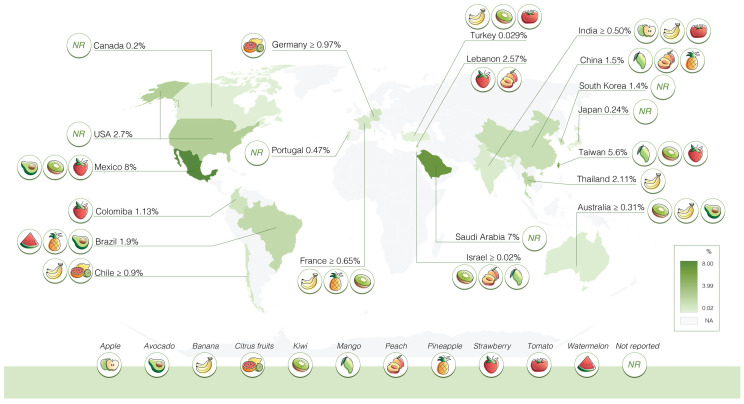
Prevalence of fruit allergy across the globe: The global prevalence of fruit allergies is derived from a systematic search spanning from 2009 to September 2023. Prevalence was either reported directly from each study or calculated as a percentage using the reported data on food or fruit allergy/hypersensitivity divided by the total study population, unless otherwise specified. In addition, the fruits featured in this figure are not an all-encompassing representation, and regional variations have been observed.

This approach yielded 45 studies with the first keyword and 6085 studies with the latter. After removing duplicates, screening abstracts, obtaining studies in English, and excluding studies conducted before the year 2009 and without a reported year of conduct, only 31 studies were eligible. Eligible studies were independently reviewed by two trained investigators (T.K. and J.N.) via the screening of full-text articles. Any disagreement during the selection process was discussed with another investigator. The global prevalence of fruit allergy and related information was independently extracted from each article by two investigators (T.K. and J.N.), and summarized in Table 1 and Figure 1. Any discrepancies during data extraction were resolved through discussion with another investigator.

Estimated fruit allergy prevalence ranges between 0.029% and 8% [1,8,10,12,13,14,18,19,21,24,25,27,30,31,32,33,35]. Ahanchian et al. and Lee et al. reported the number of fruit allergic cases as 371 and 26, respectively, without providing the total study population [11,16]. Therefore, it is not possible to estimate the prevalence of fruit allergy based on these data. The variation in study populations (community vs. hospital settings), diagnostic methods, and fruit allergy/hypersensitivity definition resulted in data heterogeneity. In 11 studies, banana was identified as one of the leading causes of fruit allergies [14,15,16,17,20,21,22,26,28,32,34], followed by kiwi in six studies [1,17,20,21,22,32], and avocado [26,28,30,32,33], mango [1,6,7,9,17], pineapple [1,7,22,30,33], and tomato [15,16,21,32,33] in five studies each. 

Regional variations were observed, with mango being predominantly reported in East Asia (particularly China and Taiwan) [1,6,7,9], and banana in Southeast Asia (Thailand) [14], South Asia (India) [15], and West Asia (Iran, Israel, Turkey) [16,17,20,21]. In Europe, only five studies have gathered data since 2009. Among these, only two studies specified types of fruit [22,23]. However, an additional study conducted across eight European countries was identified by our search strategy. Burney et al. described the prevalence of IgE sensitization in European adults [36]. This study, conducted between 2005 and 2009, did not meet our pre-specified inclusion criteria and was therefore not included in Table 1 and Figure 1. Nonetheless, it offers valuable insights into varying fruit types among these countries. In the Netherlands (Utrecht), Poland (Lodz), and Switzerland (Zurich), peach, apple, and kiwi were frequently reported. Spain (Madrid) noted peach, kiwi, and tomato allergies. In Iceland (Reykjavik), peach, kiwi, and banana allergies were prevalent, while Bulgaria (Sofia) reported tomato, banana, melon, peach, and apple allergies [36]. In Australia, the prominent fruit types were avocado and banana, which are similar to results from North and South America [26,28,30,32,33,34].

Regional variations in fruit allergy prevalence are likely influenced by dietary habits, local fruit varieties, and patterns of allergen sensitization [2,37]. Furthermore, determining the accurate prevalence of fruit allergies in the general population poses challenges due to the gold-standard diagnostic method for food allergies being the oral food challenge (OFC), which is impractical for the general population. There is also variability in how cases were defined. Some studies classified cases as individuals reporting any adverse reactions related to specific foods, while others used a validated two-step questionnaire, with the second step involving evaluations by trained healthcare staff. Some studies involved physicians’ diagnoses. More uniform and high-quality studies that use consistent diagnostic methods and definitions to determine the prevalence of fruit allergy are urgently needed.

## 3. Clinical Presentation

The allergic reaction to fruits varies in extension of symptoms, ranging from localized symptoms to multi-systems symptoms. Additionally, the severity ranges from mild to life-threatening reactions such as anaphylaxis. Details of IgE-mediated fruit allergy are summarized in Table 2. However, the most common presentation involves mild and localized symptoms known as oral allergy syndrome (OAS) [38].

### 3.1. Oral Allergy Syndrome (OAS)

Oral Allergy Syndrome (OAS), previously referred to as Plant Food Allergy Syndrome (PFAS), is commonly observed in patients presenting with symptoms primarily localized within the oral cavity in cases of cross-reactivity syndrome. The pioneering work of Tuft and Blumstein in 1942 marked the first comprehensive description of OAS, linking it to birch pollinosis and a heightened sensitivity to various fruits and vegetables [39]. Nevertheless, allergic reactions triggered by plant-based food substances extend beyond the scope of OAS. Therefore, it is essential to delineate OAS specifically to denote the swift onset of symptoms affecting the oral and pharyngeal mucosa in response to food allergens. On the other hand, the term PFAS has been introduced to characterize symptoms resulting from primary sensitization to pollen allergens, which subsequently elicit IgE-mediated cross-reactivity between aeroallergens derived from plants and plant-based food allergens, primarily associated with class 2 food allergy [39].

**Figure 2:**
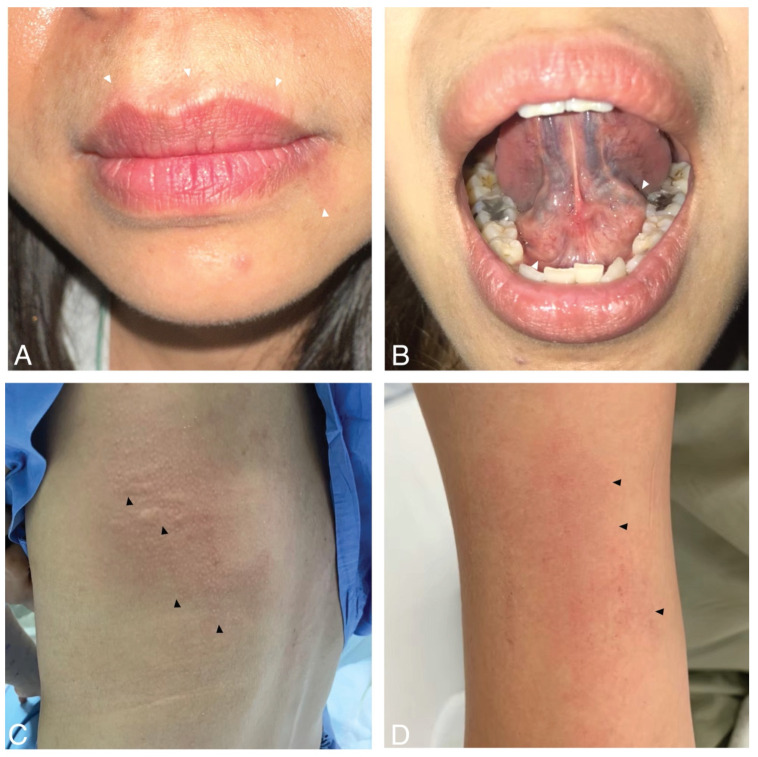
Clinical presentations of fruit allergy. (**A**,**B**) Oral allergy syndrome with perioral erythema and sublingual swelling (white arrowheads). (**C**,**D**) Systemic reactions with wheals, flare, faint erythema, and excoriations (black arrowheads).

OAS typically manifests within 2–15 min after food ingestion or the localized contact of food with the oral mucosa. These symptoms include swelling, pruritus, or numbness around the oral area, such as the lips, tongue, or palate. Figure 2A,B present a picture of a patient with OAS. While the symptoms of OAS usually remain localized to the oral area, in some cases, they can lead to systemic reactions like urticaria, rhinitis, pharyngeal edema, chest tightness, or, rarely, anaphylaxis [40,41].

### 3.2. Systemic Reaction, including Anaphylaxis

As previously noted, OAS is a typical manifestation of fruit allergies. Sometimes, systemic reactions might occur. The spectrum of systemic reactions in fruit allergy ranges from mild (generalized urticaria) to severe (anaphylaxis). Figure 2C,D present photos of urticaria and faint erythema following fruit ingestion. Systemic reactions have been documented in approximately 8.7% of cases, with severe reactions or even anaphylactic shock occurring in roughly 1.7% of instances [4]. These reactions are notably linked to several fruits, with common culprits including kiwi, banana, mango, avocado, persimmon, grape, and durian. These fruits have been identified as more likely to trigger severe allergic responses in susceptible individuals [37,42,43].

### 3.3. Food-Dependent Exercise-Induced Anaphylaxis (FDEIA)

In rare instances, the systemic reaction, food-dependent exercise-induced anaphylaxis (FDEIA), may occur. This condition is characterized by the occurrence of symptoms when specific foods are consumed in conjunction with physical exercise, typically manifesting within a time frame of 4 to 6 h following food ingestion [44]. While wheat is the most commonly implicated food allergen, there have been reports of FDEIA triggered by a variety of foods, including shrimp, shellfish, wheat, celery, tomatoes, nuts, vegetables, and fruits [45]. Furthermore, certain proteins, such as lipid transfer proteins (LTP), and the newly emergent allergen known as gibberellin-regulated protein (GRP), have also been associated with FDEIA [46]. It is noteworthy that in some cases, additional factors such as alcohol and non-steroidal anti-inflammatory drugs (NSAIDs) may contribute to the exacerbation of these reactions [47,48].

## 4. Route and Mechanism of Sensitization

The emergence of molecular allergology has significantly accelerated the investigation of both inhalant and food allergens in recent years. However, despite these advancements, the precise pathogenesis of allergic disorders remains a complex and enigmatic subject. In diverse geographical regions, various associations have been documented in relation to PFAS, involving different allergens. These associations are indicative of the influence of local aerobiology and dietary habits. We recommend adopting a molecular allergology-based perspective to enhance our understanding of PFAS, with a particular focus on elucidating clinical features, identifying patterns of clinical cross-reactivity, and potentially establishing links to the route of sensitization [39,49].

Sensitization to fruits encompasses a range of mechanisms. The primary mechanism involves direct sensitization to fruit allergens, independent of concurrent pollen allergies. The second mechanism is closely associated with respiratory pollen allergies, where fruits and pollen share structurally similar or homologous proteins, leading to cross-reactivity. This is commonly referred to as PFAS and classified as a type II food allergy. Sensitization can also occur through routes other than the respiratory and gastrointestinal tracts, such as cutaneous sensitization. A classic example of this phenomenon is sensitization to peach lipid-transfer proteins or LTPs (Pru p 3), which can occur via either cutaneous or oral exposure. Evidence for cutaneous sensitization is supported by observations of peach contact urticaria, which may even precede the onset of reactions triggered by peach ingestion [50].

Furthermore, latex fruit syndrome (LFS) is another condition that encompasses a cross-reactive syndrome primarily triggered by latex allergens, with sensitization occurring through either cutaneous or respiratory exposure. Severe allergic reactions to fruits have been associated with LFS, including bananas, avocados, chestnuts, kiwifruit, and numerous other allergenic foods [51,52]. The major immunological response to fruit allergens typically involves an IgE-mediated process, as described in reference [5]. However, in rare instances, a cell-mediated immunological mechanism has also been reported [53,54,55].

## 5. Cross-Reactivity Patterns

Allergic reactions to various fruits and vegetables can be attributed to the presence of homologous plant proteins, leading to a phenomenon known as cross-reactivity [56]. Panallergens, which are allergens with homologous IgE binding epitopes found across different species, play a crucial role in this process [57]. Table 3 provides a comprehensive summary of the cross-reactivity between fruits, vegetables, and pollen.

### 5.1. Pollen Food Allergy Syndrome (PFAS)

Pollen food allergy syndrome (PFAS) is a common condition where initial sensitization through pollen exposure leads to respiratory symptoms. Subsequent consumption of cross-reactive fruits triggers allergic reactions. Various associations have been documented in relation to PFAS. In the context of PFAS, the involved pan-allergens include various proteins such as profilin, PR-10 (pathogenesis-related protein 10), TLP (Thaumatin-like proteins), nsLTP (non-specific lipid transfer protein), GRP (gibberellin-regulated protein), seed storage proteins, cysteine protease, and β-1,3-glucanase [58].

In temperate regions characterized by notable temperature fluctuations, four distinct seasons—spring, summer, autumn, and winter—bring about varying patterns in plant pollination. Typically, trees release pollen in spring, grasses in summer, and weeds in autumn. For example, in Europe, birch trees typically begin to pollinate from March to May, while grasses usually pollinate from March to August. Ragweed, on the other hand, begins to pollinate from July to September [58,65]. The timing of pollination varies depending on the specific zone within Europe. However, certain plant species may exhibit unique pollination behaviors that diverge from these general patterns [66]. Cypress and Japanese cedar can pollinate in winter and extend to spring [58]. Table 4 represents the seasonal fluctuations in fruit pollen cross-reactivity within the context of PFAS. It is important to note that some fruits may not be harvested at the same time as the cross-reactive pollen they are associated with. For instance, cherries are typically harvested during the summer in Europe (https://www.eufic.org/en/explore-seasonal-fruit-and-vegetables-in-europe, accessed on 30 September 2023). Nevertheless, a wide variety of fruits can now be found year-round from global shipping.

#### 5.1.1. Tree Pollen

Many plant families are the sources of allergens: Betulaceae (Birch family), Oleaceae (Olive family), Platanaceae (Plane-tree family), and Cupressaceae (Cypress family). The main allergen that causes allergic reactions is the pathogenesis-related protein family 10 (PR-10) protein. PR-10 protein is a ‘pan-allergen’ found only in plant species, and is not present in animal sources. It can cause cross-reactivity with unrelated biological sources. PR-10 protein has a labile structure, similar to profilin, which is why most clinical symptoms are also mild. The most common type of pollen–fruit allergy is birch-related food allergy. The major birch allergen is Bet v1, which belongs to the PR-10 protein family. It has been found that up to 70% of Bet v1 (PR-10 protein) in the birch family is identical to other plant families. The study showed 70% of birch-sensitized patients had allergic symptoms to fruits, especially Rosaceae fruits (apple, pear, cherry, peach, plum, apricot, etc.), nuts, and vegetables, especially those from the Apiaceae family (like celery and carrots). The most common tree pollen-fruit cross-reactivity, accounting for over 75% of cases, is the Birch-apple syndrome. Most patients suffer from oral allergy symptoms. The symptom is triggered when the patient is exposed to the pollen allergen “PR-10 protein” (Bet v1 in Birch, and Mal d1 in apple). A recent study showed that sensitized birch individuals could be sensitized to apples by up to 94%, On the other hand, a sensitized apple individual could be sensitized to birch by up to 100%. Besides apple, the fruits that could be correlated with the birch pollen are peach (86%), and kiwi (28%) [3,68,69,70]. Recent reports indicate a rising prevalence of cypress sensitization in Europe and Japan, particularly among atopic individuals. These sensitization cases have been linked to various fruits, with the primary allergen remaining the PR-10 protein, resulting in OAS. However, more severe allergic symptoms have been reported in cases of peach allergy, known as Cypress-peach syndrome, often associated with the allergenic protein known as GRP [71].

#### 5.1.2. Grass Pollen

In contrast to tree and weed pollen, there are relatively limited data available on grass pollen sensitization and its association with pollen–fruit syndrome. However, historical data suggest that individuals with grass sensitization have experienced allergic reactions to a wide range of foods, including melon, watermelon, orange, tomato, potato, peanut, and Swiss chard [59]. Sensitization to grasses like Bermuda, Timothy, and Orchard grass has been linked to melon allergy, with profilin playing a role [60,61,62]. Furthermore, sensitization to orchard grass has shown associations not only with melon allergies but also with peach allergies [62].

#### 5.1.3. Weed Pollen

Most weed-causing PFAS are in the Asteraceae family, primarily including mugwort (*Artemisia vulgaris*) and ragweed (*Ambrosia artemisiifolia*). Individuals who exhibit allergies to mugwort may experience allergic symptoms upon consuming foods like carrots, celery, onion, garlic, mango, and various spices including anise, caraway, coriander, fennel, black pepper, paprika, and cumin. This interaction is mediated by a profilin called celery–mugwort–spice syndrome. Additionally, other associations have been observed, such as Asteraeae–lychee association, mugwort–peach association (nsLTP), and mugwort–chamomile association. On the other hand, ragweed cross-reacts with banana and melon via profilin and nsLTP, forming what is known as the ‘ragweed–melon–banana’ association [3,63].

### 5.2. Lipid Transfer Protein (LTP) Syndrome

Lipid transfer proteins (LTPs) are pan-allergens present in various foods and plants, including fruits, vegetables, nuts, and cereals. Sensitization to LTP can lead to symptoms in affected individuals. Some patients require co-factors such as NSAIDs, alcohol consumption, or exercise to trigger these symptoms. The most common presentation is anaphylaxis, which is more prevalent in adults and Mediterranean countries. LTPs are found in plants like mugwort, plane tree, olive, ragweed, and cypress, which are the primary sources of sensitization. Among fruits, those from the Rosaceae family are the most frequent culprits, with peach being a notable example. Clinical symptoms range from mild to severe anaphylaxis, but LTP syndrome is associated with a high incidence of anaphylaxis, affecting up to 75.6% of individuals [72,73]. 

### 5.3. Gibberellin-Regulated Protein (GRP) Syndrome

GRPs are a class of heat-stable hormones synthesized by plants in response to various stages of plant growth and development [74]. They are expressed in both the pulp and peel of fruits, with a particularly notable presence in fruits such as peach, citrus, apricot, cherry, and pomegranate [58]. In trees, GRPs are primarily found within the Cupressaceae family, which includes cypress trees [58]. Sensitization to PFAS via GRPs has been reported in southern France and Japan, and attributed to cypress and Japanese cedar, respectively [75,76]. However, patients can develop sensitization to GRPs directly through fruit exposure, independently of any cross-sensitization to tree pollens. Remarkably, 59% of patients with no prior sensitization to cypress GRP displayed sensitization to fruit GRP [39]. GRP allergies are most commonly observed in adolescents and adults, and clinical manifestations can encompass a spectrum of symptoms, including OAS, urticaria, angioedema, anaphylaxis, and FDEIA [46]. Notably, anaphylactic reactions are frequently associated with peach and apricot GRP allergies [46]. In cases of peach allergy, patients allergic to the GRP component often exhibit distinct symptoms such as facial swelling, especially in the eyelids, laryngeal tightness, and a higher prevalence of urticaria compared to patients allergic to the PR-10 component of peach [46].

### 5.4. Latex-Fruit Syndrome (LFS)

Latex is a sap derived from *Hevea brasiliensis*, containing a complex mixture of proteins, including soluble and particle-bound proteins. Some of these proteins share a structural similarity with proteins found in fruits, resulting in the presence of common antigenic determinants. This relationship has led to the recognition of a clinical syndrome known as ‘Latex-fruit syndrome’, where individuals experience allergic reactions to both latex and various fruits [64,77]. Clinical symptoms can be life-threatening, and the plant foods typically involved in the syndrome include avocado, banana, kiwifruit, and chestnut. Symptoms can vary in severity, including itching, hives, swelling, abdominal pain, vomiting, and, in more severe cases, anaphylaxis [52,77].

Among NRL (natural rubber latex) allergens, class 1 chitinases (Hev b 6) play a significant role in the LFS. Class 1 chitinases have a defensive function, and Hev b 6 exhibits high sequence homology with chitinases found in fruits like bananas, avocados, and chestnuts [78]. Other significant NRL allergens include β-1,3-glucanase (Hev b 2), found in various fruits such as avocado, banana, chestnut, fig, kiwi, and olive pollen, as well as the acidic protein (Hev b 5), which has the potential for cross-reactivity with kiwi acid protein. In a retrospective study involving 137 patients with a history of natural rubber allergy and positive latex tests, symptoms were reported upon exposure to a range of fruits, including banana, avocado, kiwi, tomato, watermelon, peach, chestnut, cherry, apple, apricot, and strawberry [79].

Brehler et al. (1997) conducted a study demonstrating that the levels of latex-specific IgE were inhibited when serum samples from individuals with latex allergies were preincubated with extracts from specific fruits. Fruits such as avocado, banana, fig, tomato, kiwi, melon, and passion fruit were found to be particularly effective in inhibiting latex-specific IgE levels [51]. Nevertheless, there are still groups of patients who exhibit sensitization to specific fruits independently of NRL sensitization. This suggests that there are responsible allergens for fruit allergies in specific regions or cases [37].

### 5.5. Thaumatin-Like Proteins (TLP) Syndrome

Thaumatin, found in the fruits of the West African rainforest shrub *Thaumatococcus daniellii*, shares sequence homology with PR-5 proteins and imparts a sweet taste. These proteins belong to the family known as thaumatin-like proteins (TLPs). TLPs are known for their resistance to proteases and resistance to changes in pH or heat. They respond to pathogen infection, osmotic stress (osmotins), and antifungal proteins [80] Both TLPs and PR-5 are expressed in ripening fruits [58]. TLPs serve as allergenic molecules in many fruit allergies including apple, banana, cherry, kiwi, and peach [58], and might also be a causative allergen in patients with orange-dependent exercise-induced anaphylaxis [81]. In mouse models, there are reports of percutaneous sensitization to TLP. Given that cherries are widely utilized in cosmetics, including lip care products, this raises the potential for percutaneous sensitization in humans [82]. TLPs are prevalent in numerous fruits and can act as pan-allergens, provoking allergic reactions to a variety of fruits. Nonetheless, data on cross-reactivity patterns and clinical severity are limited. 

## 6. Management

Allergic reactions to fruits, as with other food allergies, can have a multifaceted influence on an individual’s health. Such allergies mandate that individuals not only avoid the inciting fruits but also exercise caution due to the potential for cross-reactivity with other fruits. Compounding this challenge is the ubiquitous presence of fruits in numerous products ranging from juices and pastries to preserves, sweets, and various culinary dishes.

Fruits are universally acknowledged for their rich contents of vitamins, minerals, and dietary fiber [83]. For those who are allergic to multiple fruits, the burden is on identifying alternative sources for these vital nutrients. Such individuals may benefit substantially from dietary counseling to facilitate the formulation of a holistic and nutritious diet [84]. Furthermore, individuals living with fruit allergies often struggle with increased anxiety, stress, and fear of unintentional exposure. This can profoundly influence their emotional health, daily activities, and social engagements, especially when eating out, partaking in social gatherings, or traveling [85]. The escalating prevalence of fruit allergies, combined with the complexities of cross-reactivity, could instigate transformative shifts in the food supply chain. This might include diminished production of certain fruits known for allergenicity, modifications in agricultural practices, augmented demand for allergen-free alternatives, and economic repercussions. Changes in food processing and a potential alteration in biodiversity within the food ecosystem may also arise as indirect consequences [86].

Fruit allergies exhibit a wide range of clinical manifestations, from mild oral symptoms to severe anaphylaxis, setting them apart from other food allergies. Cross-reaction patterns, which vary based on molecular sensitization and regional factors, along with a lack of robust evidence, contribute to diverse recommendations [58]. In suspected cases, patients should undergo evaluation by an allergist. For anaphylactic or respiratory symptoms, strict avoidance and cross-reactivity assessments are advised. In milder cases with oral symptoms, heated fruit consumption may be considered based on skin tests, after careful risk–benefit analysis and patient consent.

The general concept for managing food allergies encompasses four key components: (1) allergen avoidance, including consideration of cross-reactivity; (2) carrying prefilled epinephrine syringes/autoinjectors; (3) possessing an action plan; (4) and accessing advanced treatment for food allergies [2]. There is no evidence-based advice about fruit avoidance. The survey conducted in the United States among allergists from the American Academy of Allergy, Asthma, and Immunology (AAAAI) directory showed different answers ranging from complete avoidance to no restriction, but a majority of allergists prefer complete avoidance or personalized management [87]. 

In the recent 2022 European Association of Allergy & Immunology (EAACI) recommendations, precise patient diagnosis is emphasized, categorizing allergies into distinct phenotypic patterns. These patterns include Pattern A, characterized by sensitization to Bet v 1 or Bet v 1 homologues; Pattern B, involving sensitization to nsLTP; and Pattern C, indicating sensitization to profilin, which may lead to cross-sensitization with profilin in Rosaceae fruits. Additional patterns are outlined for specific fruits, such as kiwi. Pattern D encompasses latex protein sensitization, potentially leading to cross-sensitization to kiwi proteins, with clinical presentations varying from mild OAS to anaphylaxis. In Pattern E, patients exhibit monosensitization to a specific fruit protein, such as Act d 1 in kiwi cases, which carries an increased risk of systemic reactions [58].

### 6.1. Avoiding Fruit Allergens

According to Food Allergy: A Practice Parameter, 2014, from the AAAAI, dietary avoidance of raw fruits and vegetables is based on the patient’s symptom severity [88]. Personalized treatment for each patient may be the best approach, because many factors involving severity include part of the fruit, amount of fruit, type of allergen sensitization, heat processing or not, and cofactor (exercise, alcohol, NSAIDs, antiacids) [58]. 

As per the EAACI’s 2022 recommendations, the avoidance of raw fruits that trigger symptoms is advised across all patterns. However, the guidance regarding processed fruits varies. In Pattern A/C, processed foods should be avoided if there are positive oral challenges or if they have been reported to elicit symptoms. For Patterns B/D/E, it is recommended to avoid both the raw fruits that cause symptoms and processed fruits [58].

Consider a patient who feels itching in the mouth and has swollen lips after eating an apple. He also has a history of seasonal allergic rhinitis to birch. The doctor believes the patient might be allergic to apples (manifesting as OAS) with potential cross-reactivity to other fruits. The patient is subsequently advised to avoid apples and any fruits he has not previously consumed, and to pursue a comprehensive evaluation by an allergist. A blood test called serum-specific IgE testing shows a reaction to Mal d 1 (related to Bet v 1), but no reactions to other tested apple components, including nsLTP (Mal d 3). Based on this molecular allergy diagnosis, it is inferred that the patient has a birch-related PFAS or a Bet v 1-related fruit/vegetable allergy (Pattern A), with no evidence of nsLTP sensitization. This means the patient is likely to have a mild reaction (OAS) with a small chance of a severe reaction. Symptoms, it is observed, are predominantly elicited by unprocessed foods. Patients generally tolerate processed fruits. Although in general, patients usually tolerate processed fruit, Bet v 1 (PR-10) related allergies can be associated with systemic reactions in the presence of co-factors, ingestion on an empty stomach, and consuming a high quantity of fruits. Thus, the recommendation is to avoid consuming raw apple items, including fresh apples and apple juice, and to avoid any fruits or vegetables previously associated with reactions, such as pear, cherry, peach, plum, apricot, celery, carrot, potato, and certain nuts. Processed fruits or vegetables need only be avoided if they have triggered a reaction during oral food challenges. Co-factors like exercise, NSAIDs, and alcohol can intensify the reaction. In addition, ingestion on an empty stomach and consuming a high quantity can also be associated with a systemic reaction.

However, some countries and facilities lack access to precise diagnostics at the allergen component level. This limitation complicates predicting cross-reactivity patterns with other fruits and forecasting the severity of future reactions. While the oral food challenge remains the gold standard for diagnosing fruit allergies, it carries a risk of systemic reactions and can be impractical due to the associated medical expenses. To tackle this issue, the availability of component-resolved diagnosis (CRD) for molecular diagnosis is crucial. Additionally, the development of less invasive and more accurate in vitro tests to confirm fruit allergy diagnoses would be immensely beneficial.

### 6.2. Medications

While most clinicians believe that PFAS does not cause anaphylaxis [63], there is increasing evidence of reported anaphylaxis [43,89]. Due to a lack of high-quality evidence on the recommendation of epinephrine autoinjectors, clinicians should be aware that fruit allergens can cause anaphylaxis, and should prescribe epinephrine autoinjectors to high-risk patients based on their previous reactions. Moreover, the risk of anaphylactic reactions may be associated with specific fruits. The common fruits causing anaphylaxis may vary between regions. In North America, the most common fruits causing anaphylaxis were kiwi, banana, and mango [43], whereas in South Korea, the most common anaphylaxis-triggering fruits in PFAS were apple and peach [89]. Other factors that may be associated with anaphylaxis are concomitant eczema and the spring season [43,89]. 

In contrast, as per the EAACI 2022 recommendations, for Patterns A/C, it is recommended to self-administer emergency medication orally due to the relatively small risk of systemic reactions or severe local reactions. This typically involves taking antihistamines and, if necessary, steroids. On the other hand, for Patterns B/D/E, it is recommended to use oral medications as the first-line treatment, and individuals should also carry self-injectable epinephrine in case they experience a systemic reaction [58].

### 6.3. Novel Therapies

There is currently no proven cure for patients with fruit allergies. However, in certain cases, such as food allergies, studies have explored oral immunotherapy as a potential treatment option. For example, a study was conducted that specifically focused on oral immunotherapy using peach juice. This study involved 24 patients who were allergic to LTP, and aimed to evaluate the effectiveness of this approach in managing their allergies [90]. By the end of the study, it was found that 17 out of the 24 patients were able to tolerate 200 mL of peach juice, indicating a positive response to the oral immunotherapy approach. Another investigation explored the use of sublingual immunotherapy (SLIT) with peach extract as a potential treatment for fruit allergies [91]. Following 6 months of sublingual immunotherapy (SLIT), patients demonstrated increased tolerance to peach, with their tolerance levels ranging from three to nine times higher than before treatment. Additionally, there was a significant reduction (5.3 times) in skin prick test (SPT) reactions and IgG responses among these patients. It is worth noting that mild oral reactions were observed in the SLIT group, which suggests that while SLIT can improve tolerance, some patients may still experience mild allergic reactions during treatment.

In a separate study conducted by Kopac in 2012, individuals with OAS related to apples underwent oral immunotherapy (OIT). In this OIT regimen, participants were administered 128 g of apple over 8 months as part of their treatment plan [92]. In a study involving oral immunotherapy (OIT) for individuals with OAS related to apples, the OIT group experienced a more significant decline in their allergy symptoms when compared to the placebo group. This suggests that OIT may be an effective approach for managing OAS in individuals with apple allergies.

Additionally, there have been case reports of using omalizumab to treat latex fruit syndrome, a condition wherein individuals with latex allergies experience cross-reactivity with certain fruits. In these cases, omalizumab treatment resulted in improved asthma control and reduced lip edema in patients with pollen food syndrome. Omalizumab is a monoclonal antibody used to treat allergic conditions, and has shown promise in managing allergies related to latex fruit syndrome when traditional treatments may be insufficient [93,94,95]. However, it is important to note that overall, these studies have involved a limited number of participants and require further investigation.

## 7. Conclusions

In conclusion, we provide valuable insights into the complex landscape of fruit allergies. The prevalence of fruit allergies varies across regions, influenced by factors such as local aerobiology and dietary habits. Clinical presentations range from mild symptoms such as OAS to severe systemic reactions, including anaphylaxis, emphasizing the importance of accurate diagnosis and management. The review highlights the diverse mechanisms of sensitization to fruit allergens, including direct sensitization, cross-reactivity with respiratory pollen allergens, and even cutaneous sensitization.

Effective management of fruit allergies necessitates a personalized approach, considering factors like symptom severity, specific allergen sensitization, heat processing, and co-factors like exercise, alcohol, and NSAIDs. The review discusses the importance of allergen avoidance, the availability of emergency medications like epinephrine autoinjectors, and the potential for novel therapies like oral immunotherapy and omalizumab. While there is no cure for fruit allergies, ongoing research offers hope for improved treatments and a better quality of life for affected individuals. Overall, this review contributes to our understanding of the multifaceted nature of fruit allergies and underscores the need for individualized care and ongoing research in this field.

## Figures and Tables

**Table 1:** Characteristics and prevalence of fruit allergy of eligible studies. (N = 31 studies).

Study (Year)	Country	Setting	Age (y, Mean ± SD)	Total Study Population	Data Source	Diagnostic Test	Prevalence of Fruit Allergy	Leading Types of Fruit (N, % of Total Study Population)
**East Asia**								
Feng (2023) [6]	China (Jiangxi)	Community-based	44.72 ± 12.91	11,935	Self-reported	NA	NR	- Mango (89, 0.75%)
Feng (2022) [7]	China (Jiangxi)	Community-based	8.67 ± 1.26	8856	Parent-reported	NA	NR	- Mango (165, 1.86%) - Peach (5, 0.06%) - Pineapple (5, 0.06%)
Sha (2019) [8]	China (Beijing)	Community-based	0–14 *	13,073	Parent-reported	NA	1.5%	NR
Zeng (2015) [9]	China (Guangdong)	Community-based	4.6 ± 1.1	2540	Parent-reported	NA	NR	- Mango (58, 2.3%)
Kaneko (2015) [10]	Japan (Kawasaki)	Community-based	0–5 *	23,969	Physician-diagnosed	NR	0.24%	NR
Lee (2017) [11]	South Korea (Suwon)	Hospital-based	38.5 ± 14.1	95 ^†^	Physician-diagnosed	SPT, sIgE	26 ^¶^	NR
Kim (2017) [12]	South Korea (Nationwide)	Community-based	6–16 *	29,842	Parent-reported	NA	1.41%	NR
Su (2023) [13]	Taiwan (Taipei)	Community-based	6–7 *, 13–14 *, and Adult [Median (IQR) 42 (39–46)]	16,200	Parent-reported and self-reported	NA	6–7 y: 0.96% 13–14 y: 1.16% Adult: 0.79% Overall: 0.89%	NR
Li (2022) [1]	Taiwan (Nationwide)	Community-based	Mean (range) 10.1 (6–13)	9982	Parent-reported and self-reported	NA	5.6%	- Mango (3.6%) - Kiwi (1.3%) - Strawberry, Orange, Pineapple, Watermelon (0.5% each)
**Southeast Asia**								
Sompornrattanaphan (2023) [14]	Thailand (Bangkok)	Hospital-based	Median (IQR) 31.0 (24.0, 44.0)	711	Physician-diagnosed	SPT, sIgE, OFC	2.11%	- Banana (unpublished data)
**South Asia**								
Mahesh (2016) [15]	India (Bangalore, Mysore)	Community-based	20–54 *	10,931	Self-reported and physician-diagnosed	sIgE	NR	- Apple (0.50%) ‡ - Banana (0.05%) ‡ - Tomato (0.02%) ‡
**West Asia**								
Ahanchian (2016) [16]	Iran (Khorasan)	Hospital-based	Mean(range) 5.34 (0–18)	NR	Physician-diagnosed	SPT	371 ^¶^	- Tomato (7.8%) § - Orange (4.7%) § - Banana (4%) § - Grape (3.7%) §
Nachshon (2019) [17]	Israel (Recruitment center of the Israel Defense Forces)	Community-based	17	12,592	Self-reported and physician-diagnosed	SPT, OFC	NR	- Kiwi, Peach (3, 0.024% each) - Mango (2, 0.016%) - Apple, Banana, Fig, Plum, Strawberry (1, 0.008% each)
Irani (2015) [18]	Lebanon	Community-based	NR (infants, children, and adults)	506	Self-reported	NA	2.57%	- Strawberry (6, 1.19%) - Peach (5, 0.99%)
Alotiby (2022) [19]	Saudi Arabia (Makkah)	Community-based	18–80 *	531	Self-reported	NA	7%	NR
Akarsu (2021) [20]	Turkey (Ankara)	Hospital-based	Median (IQR) 6 (5–7.63)	534^†^	Physician-diagnosed	SPT, sIgE, OFC	NR	3–5 y (n = 440): - Kiwi (6) - Banana (2) 6–12 y (n = 217): - Kiwi (7) - Banana (2) 13–18 y (n = 35): - Banana (1)
Kaya (2013) [21]	Turkey (Ankara)	Community-based	12.9 ± 0.9	10,096	Parent-reported and physician-diagnosed	SPT, sIgE, OFC	0.029%	- Banana, Kiwi, Tomato (1, 0.001% each)
**Europe**								
Tamazouzt (2022) [22]	France (Nationwide)	Community-based, birth cohort	0–5.5 *	16,400	Parent-reported	NA	NR	- Exotic fruit (i.e., banana, pineapple, and kiwi, 0.65%) ‡
Röhrl (2022) [23]	Germany	Community-based	0–2 *	KUNO Kids cohort 1139	Parent-reported	NA	NR	- Citrus fruit 0.97%
				SPATZ cohort 1006	Physician-diagnosed	NR	NR	NR
Lozoya-Ibáñez (2020) [24]	Portugal (Central region)	Community-based	14.3 ± 1.1	1702	Self-reported and physician-diagnosed	SPT, sIgE, OFC	0.47%	NR
Lozoya-Ibáñez (2016) [25]	Portugal (Central region)	Community-based	Mean(range) 48 (18–80)	965	Self-reported and physician-diagnosed	SPT, sIgE, OFC	0.1%	NR
**Oceania**								
Sasaki (2017) [26]	Australia	Community-based	10–14 *	9816	Parent-reported and Physician-diagnosed	SPT, sIgE, OFC	NR	Clinic group ^※^ - Kiwi (7, 0.1%) - Avocado (3, 0.05%) Self-reported FA ^※^ - Kiwi (23, 0.5%) - Banana (7, 0.2%) - Avocado (1, 0.02%)
**North America**								
Singer (2021) [27]	Canada	Hospital-based (Primary care)	≤19	288,490	Physician-diagnosed	NR	0.2%	NR
Bedolla-Pulido (2019) [28]	Mexico (Guadalajara)	Community-based	15–18 *	1992	Self-reported	NA	NR	- Avocado (1.0%) - Banana (0.8%) - Peach, Apple (0.7% each)
Ontiveros (2016) [29]	Mexico (Culiacan)	Community-based	8.6 (5–13)	1049	Parent-reported	NA	NR	- Strawberry (8, 0.76%) - Citric fruits (2, 0.19%)
Puente-Fernández (2016) [30]	Mexico (Toluca)	Community-based	18–25	1200	Self-reported	NA	8%	- Avocado, Kiwi (17, 1.4% each) - Pineapple (16, 1.3%) - Guava (12, 1.0%)
Verril (2015) [31]	United States	Community-based	≥18	4568	Self-reported	NA	2.7% ^‡^	NR
**South America**								
da S. Correia (2022) [32]	Brazil (Limoeiro town, Pernambuco state)	Community-based	3.6 ± 1.1	412	Parent-reported	NA	1.9%	- Coconut (2, 0.48%) - Acai, Apple, Avocado, Banana, Guava, Tomato (1, 0.24% each)
Silva (2016) [33]	Brazil (Uberlandia)	Community-based	18–65 *	1583	Self-reported	NA	1.6%	- Watermelon - Pineapple - Avocado - Tomato
Hoyos-Bachiloglu (2014) [34]	Chile (Santiago)	Community-based	~5, 10, 15	455	Parent-reported	NA	NR	- Banana (0.9%) - Citrus fruits (0.4%)
Beltrán-Cárdenas (2021) [35]	Colombia (Medellín)	Community-based	5–12 *	969	Self-reported	NA	1.13%	- Strawberry (7, 0.72%) - Other fruits were not mentioned.

Note: Studies were stratified by continents and arranged according to alphabetical order of country’s name, and year of publication. Prevalence was either reported directly from each study or calculated as a percentage using the reported data on food or fruit allergy/hypersensitivity divided by the total study population, unless otherwise specified. Abbreviations: FA, food allergy; IQR, interquartile range; NA, not available; NR, not reported; OFC, oral food challenge; SD, standard deviation; sIgE, serum specific immunoglobulin E; SPT, skin prick test; y, year. * Range; ¶ Number of fruit-allergic patients; † Number of food-allergic patients; § Percentage per food-allergic patients; ‡ Weighted prevalence; ※ Clinic group (n = 5016) consisted of students who had a parent-reported questionnaire, with successful phone contact and completion of clinic evaluation. Self-reported group (n = 4800) consisted of the remaining students, who had a student questionnaire only or parent-reported questionnaire but without nurse contact or completion of clinic evaluation.

**Table 2:** Details of clinical presentations of IgE-mediated fruit allergy.

Clinical Patterns	Area of Involvement	Onset	Clinical Characteristics
**Local reaction**			
Oral allergy syndrome (OAS)	Localized to oral area	Within a few minutes (2–15 min) after exposure via direct contact	- Initial sensitization to pollen allergens can lead to IgE-mediated cross-reactivity with plant-food allergies. - Manifestations such as swelling, pruritus, or numbness are often confined to the oral region, specifically the lips, tongue, or palate upon direct exposure to fruit allergens. - OAS can present as an isolated phenomenon or as preceding symptoms of systemic allergic reactions.
**Systemic reaction**			
Non-anaphylactic systemic reaction	Systemic manifestations	Within 2–3 h	- The reaction can affect any organ system. However, it is predominantly cutaneous, manifesting as urticaria and/or angioedema following plant food consumption away from the direct contact site. This reaction does not meet the criteria for anaphylaxis.
Anaphylaxis	Systemic manifestations	Within 2–3 h	- A severe, life-threatening reaction can affect multiple organ systems or result in profound impairment of a single organ system.
Food-dependent exercise-induced anaphylaxis (rare)	Manifestations of symptoms associated with exercise or other cofactors	Within 4–6 h	- Anaphylaxis can be triggered when specific foods are consumed in conjunction with physical exertion or other cofactors (such as alcohol or certain drugs), typically manifesting within 4 to 6 h. - Consuming these specific foods does not trigger symptoms without physical exertion or cofactors.

**Table 3:** Fruit and pollen cross-reactivity within the context of pollen food allergy syndrome (PFAS).

Primary Pollen Sensitization	Fruit	Vegetable	Others
Birch [3]	Apple, pear, cherry, peach, plum, apricot	Celery, carrot, potato	Hazelnut, peanut, soy
	Kiwi		
Cypress, Japanese Cedar [58]	Peach, citrus, apricot, cherry, pomegranate		
Grass (Bermuda, Orchard, Timothy) [59,60,61,62]	Cantaloupe, honeydew, watermelon	Potato	
	Orange, peach		
	Tomato		
Mugwort [3,63]		Celery, carrot, garlic, onion, parsley, bell pepper, broccoli, cabbage, cauliflower, chard	Spice (anise, caraway, coriander, fennel, black pepper, paprika, cumin)
			Chamomile Sunflower
Ragweed [3,63]	Cantaloupe, honeydew, watermelon	Zucchini	
	Banana		
Latex [64]	Banana		
	Kiwi		
	Avocado		
	Chestnut		

**Table 4:** Seasonal variation of fruit–pollen cross-reactivity in pollen food allergy syndrome (PFAS).

	Spring	Summer	Fall	Winter
Apple	√			
Apricot	√			√
Cherry	√			√
Peach	√	√		√
Pear	√			
Plum	√			
Cantaloupe		√	√	
Honeydew		√	√	
Watermelon		√	√	
Banana			√	
Kiwi	√			
Orange		√		√
Tomato		√		
Pomegranate				√

Adapted with permission from Ref. [67]. Copyright 2020 American Academy of Allergy, Asthma and Immunology.

## Data Availability

Not applicable.
